# Supplementary material for: Cystic proliferation of germline stem cells is necessary to reproductive success and normal mating behavior in medaka
Source: eLife. 2021 Mar 1;10:e62757. doi: 10.7554/eLife.62757 (PMC7946426; doi:10.7554/eLife.62757)
Supplement: Supplementary file 1. [file elife-62757-supp1.docx]

**Supplementary file 1A.** Primers sequences, ENSEMBL accession numbers and respective references of each gene were added.

| **Gene symbol** | **Accession Number** | **primer sequence (5´- 3´)** | **Experiment** |
| --- | --- | --- | --- |
| ***ndrg1a*** | ENSORLG00020002065 | **Fw:** GACGATATCCAGGTTGTCGAGTCC **Rv:** CGATGATATGGTTGCGATATCTCGC | **WMISH** |
| ***ndrg1b*** | ENSORLG00020022631 | **Fw:** CATGTTGAGGCTCCAGGACAAC **Rv:** CTGCAGCTCGTTGGTATGTGAG |  |
| ***oct4*** | ENSORLG00000010471.2 | **Fw:** GGCCCCGGCTTTCTTTG **Rv:** CTCCGCTTCCTCTTCCTGGTG |  |
| ***ndrg1b*** | ENSORLG00020022631 | **Fw:** ATGTCAACCCCAATGCTGAG **Rv:** CGTTGGACTGGTTCATGGTT | **RT-qPCR** |
| ***RPL7*** | ENSORLG00000007967.2 | **Fw:** CGCCAGATCTTCAACGGTGTAT **Rv:** AGGCTCAGCAATCCTCAGCAT |  |
| ***ef1*α** | ENSORLG00000007614 | **Fw:** GGAGGCCAGCGACAAGATGAGC.  **Rv:** ACACGGCCGACAGGGACAGTTC |  |
| ***dmy*** | ENSORLG00000020486 | **Fw:** CAACTTTGTCCAAACTCTGA **Rv:** TGATGCAGCATTTTGACACATTTA | **Sexing PCR** |
| ***B-actin*** | ENSORLG0000001367 | **Fw:** GGATGACATGGAGAAGATCTGG **Rv:** ATGGTGATGACCTGTCCGTC-3’ |  |
| ***ndrg1b*** | ENSORLG00000004785 | **Fw:** CTTGACATGCCTTTATCTAGAGAC **Rv:** CATTTCTGTCACCAGCTTAAG | **HMA** |
| ***Chr1*** | chr1:5601523-5601805 | **Fw:** GGGTGAATGTTGCAGAAGTTG **Rv:** GGACTGTTGGAATGTGGGTG | **Off-target HMA** |
| ***Chr3*** | chr3:4654886+4655208 | **Fw:** CTCTGTGGTCCAGTACCAACTG. **Rv:** GGTGAGTTGATAATGCGGTC |  |
| ***Chr7*** | chr7:21163167+21163418 | **Fw:** GGAGCCGCCTGTTAGCTTC **Rv:** CTGGCTCGTGTGACACATACG |  |

**Supplementary file 1B.** Sex ratio of both sexes embryos injected with *cas9* (wildtype) and the sg*Nb1* (cas9+sg1_*ndrg1b*).

| **Sex ratio** | | | | | |
| --- | --- | --- | --- | --- | --- |
|  | **wt** | | **sg*N1b*** | | **% sex reversal** |
|  | **Testis** | **Ovary** | **Testis** | **Ovary** |  |
| **Female (XX)** | 0 | 26 | 2 | 21 | 8,70 |
| **Male (XY)** | 30 | 0 | 28 | 1 | 3,45 |
