# Supplementary material for: Cystic proliferation of germline stem cells is necessary to reproductive success and normal mating behavior in medaka
Source: eLife. 2021 Mar 1;10:e62757. doi: 10.7554/eLife.62757 (PMC7946426; doi:10.7554/eLife.62757)
Supplement: Transparent reporting form [file elife-62757-transrepform.docx]

***eLife’s* transparent reporting form**

We encourage authors to provide detailed information *within their submission* to facilitate the interpretation and replication of experiments. Authors can upload supporting documentation to indicate the use of appropriate reporting guidelines for health-related research (see [EQUATOR Network](http://www.equator-network.org/%20)), life science research (see the [BioSharing Information Resource](https://biosharing.org/)), or the [ARRIVE guidelines](http://www.plosbiology.org/article/info:doi/10.1371/journal.pbio.1000412) for reporting work involving animal research. Where applicable, authors should refer to any relevant reporting standards documents in this form.

If you have any questions, please consult our Journal Policies and/or contact us: [editorial@elifesciences.org](mailto:editorial@elifesciences.org).

**Sample-size estimation**

- You should state whether an appropriate sample size was computed when the study was being designed
- You should state the statistical method of sample size computation and any required assumptions
- If no explicit power analysis was used, you should describe how you decided what sample (replicate) size (number) to use

Please outline where this information can be found within the submission (e.g., sections or figure legends), or explain why this information doesn’t apply to your submission:

The sample-size of all experiments are specified in the legend of each figure.

**Figure 1:** RNA quantification by RT-qPCR: n= 5-6 pools of 5 individuals per sex in stage 35 and 39; n= 6-7 individuals per sex in 10 and 20 dph

**Figure 2:** germ cells quantification at stage 39: n=8 individuals per each wt group and n=12 individuals per each sgN1b group.

**Figure 3:** germ cells quantification at 20 dph: n=16 individuals per each wt and sgN1b female group and n=20 individuals per each wt and sgN1b male group.

**Figure 4:** reproductive success: n=10 couples per each group. Germ cells quantification at 80 dph: n=10 individuals per each female and male group.

**Figure 5:** sexual behavior: n=5 couples per each group.

**Figure S1:** germ cells quantification at stage 39: n=5 individuals per each group. RNA quantification by RT-qPCR: n= 5 pools of 5 individuals per group.

**Figure S2:** mutation percentage of sg1_*ndrg1b*: n=20 individuals, sequencing *at F1*: n=18 individuals, off-targets mutation percentage: n=5 individuals.

**Figure S3:** mutation percentage of sg2_*ndrg1b*: n=20 individuals, germ cells quantification at stage 39: n=3 individuals per each group.

**Figure S4:** TUNEL-positive germ cells quantification at stage 39: n=4 individuals per each group.

**Replicates**

- You should report how often each experiment was performed
- You should include a definition of biological versus technical replication
- The data obtained should be provided and sufficient information should be provided to indicate the number of independent biological and/or technical replicates
- If you encountered any outliers, you should describe how these were handled
- Criteria for exclusion/inclusion of data should be clearly stated
- High-throughput sequence data should be uploaded before submission, with a private link for reviewers provided (these are available from both GEO and ArrayExpress)

Please outline where this information can be found within the submission (e.g., sections or figure legends), or explain why this information doesn’t apply to your submission:

The information about biological and technical replicates of all experiments are specified in each section of materials and methods.

**RNA quantification by RT-qPCR:** biological replicates correspond with each individual and pool of individuals. 2 technical replicates were used per each gene in each RT-qPCR assay. Samples with amplifications with primer dimers were excluded of all analysis.

**CRISPR/Cas9 to generate *ndrg1b* mutants:** biological replicates correspond with all injected individuals used to biallelic mutations and off-target analysis. Technical replicates correspond to both target sites designed and analyzed.

**Immunofluorescence, TUNEL and histological analysis:** biological replicates correspond with each individual. All experiments were performed at 3 different times with different biological replicates always including both sexes, controls and mutants.

**Germ cell quantification:** biological replicates correspond with each individual analyzed. For stage 39 individuals were counted all gonadal sections. For 20 and 80 dph individuals were counted three gonadal sections figuring the distance between each section to avoid count the same cell twice. 2 technical replicates were used per each counted section.

**Sperm quantification:** biological replicates correspond with each individual analyzed. Sperm collection was performed at 2 different times with different biological replicates including controls and mutants. 3 technical replicates were used per each counting and individual.

**Evaluation of reproductive success:** biological replicates correspond with each couple analyzed. Evaluation was performed at 2 different times with different biological replicates including all categories analyzed.

**Sexual behavior assay:** biological replicates correspond with each couple analyzed. The fish tanks were randomly distributed on the rack. Females that did not spawn or spawned without any mating behavior were excluded from the analysis. The analysis of the videos was performed twice by two different authors of the present study who did not know the category of the couple (double-blind experiment).

**Statistical reporting**

- Statistical analysis methods should be described and justified
- Raw data should be presented in figures whenever informative to do so (typically when N per group is less than 10)
- For each experiment, you should identify the statistical tests used, exact values of N, definitions of center, methods of multiple test correction, and dispersion and precision measures (e.g., mean, median, SD, SEM, confidence intervals; and, for the major substantive results, a measure of effect size (e.g., Pearson's r, Cohen's d)
- Report exact p-values wherever possible alongside the summary statistics and 95% confidence intervals. These should be reported for all key questions and not only when the p-value is less than 0.05.

Please outline where this information can be found within the submission (e.g., sections or figure legends), or explain why this information doesn’t apply to your submission:

Statistical reporting of each experiment is specified in the legend of each figure and in the section of statistical analysis in materials and methods.

Continuous variables were compared between two groups by the unpaired two-tailed Student’s t-test. If the F-test indicated that the variance differed significantly between groups, Welch’s correction to the Student’s t-test was employed. For more than two groups, continuous variables were compared by one-way analysis of variance (ANOVA), followed by Dunnett’s post hoc test, for comparisons of experimental groups versus control group). All differences were considered statistically significant when P<0.05.

(For large datasets, or papers with a very large number of statistical tests, you may upload a single table file with tests, Ns, etc., with reference to sections in the manuscript.)

**Group allocation**

- Indicate how samples were allocated into experimental groups (in the case of clinical studies, please specify allocation to treatment method); if randomization was used, please also state if restricted randomization was applied
- Indicate if masking was used during group allocation, data collection and/or data analysis

Please outline where this information can be found within the submission (e.g., sections or figure legends), or explain why this information doesn’t apply to your submission:

The information about group allocation are specified in materials and methods.

All biological samples were processed and analyzed in random.

**Additional data files (“source data”)**

- We encourage you to upload relevant additional data files, such as numerical data that are represented as a graph in a figure, or as a summary table
- Where provided, these should be in the most useful format, and they can be uploaded as “Source data” files linked to a main figure or table
- Include model definition files including the full list of parameters used
- Include code used for data analysis (e.g., R, MatLab)
- Avoid stating that data files are “available upon request”

Please indicate the figures or tables for which source data files have been provided:

**Figure S1:** TGF-β is not involved in the *ndrg1b*-mediated cystic proliferation in females during early gonadal development.

**Figure S2:** CRISPR/Cas9: sg1_*ndrg1b* design, heteroduplex mobility assay (HMA), efficiency and potential off-targets.

**Figure S3:** Corroboration with a second RNA guide (sg2_*ndrg1b*) the specificity of CRISPR/Cas9 methodology to mutate *ndrg1b*.

**Figure S4:** Apoptosis of EGSCs at stage 39

**Table S1.** Primers sequences, ENSEMBL accession numbers and respective references were added to each gene

**Table** **S2.** Sex ratio of both sexes embryos injected with cas9 (wild type) and the sgNb1 (cas9+sg1_ndrg1b).
